# Calcium Homeostasis and Parturient Paresis in Ruminants: Mechanistic Insights and Clinical Management

**DOI:** 10.3390/ani16162597

**Published:** 2026-08-19

**Authors:** Meiqiang Chu, Yanan Wang, Zhennan Wang, Shenjin Lv

**Affiliations:** 1 College of Agriculture and Forestry Science, Linyi University, Linyi 276000, China; chumeiqiang@lyu.edu.cn (M.C.); 19709850212@163.com (Y.W.); 2 Key Laboratory of Animal Genetics, China Agricultural University, Beijing 100193, China

**Keywords:** parturient paresis, calcium homeostasis, prevention strategies

## Abstract

Parturient paresis remains a major economic challenge in global ruminant production, causing acute periparturient hypocalcemia and predisposing high-yielding animals to a cluster of secondary pathologies. Traditional narratives often treat regulatory pathways in isolation, whereas this review synthesizes multi-organ endocrine networks to address the kinetic dyssynchrony between mammary calcium drain and homeostatic recruitment velocity. Beyond the classical parathyroid hormone–vitamin D axis, we integrate the mammary–gut–bone axis into a unified endocrine model, highlighting the critical role of the serotonin–parathyroid hormone-related protein rheostat for skeletal mineral mobilization and the fibroblast growth factor 23–Klotho axis in prepartum phosphorus-induced feedback suppression. We evaluate the molecular mechanisms underlying target-organ receptor resistance, driven by vitamin D receptor downregulation and epigenetic aging, which precipitate homeostatic feedback failure. Regarding clinical management, this synthesis contrasts reactive parenteral interventions with proactive nutritional priming strategies, such as negative dietary cation–anion difference acidification, zeolite-based gastrointestinal binders, and exogenous vitamin D or 5-hydroxytryptophan supplementation. Additionally, the role of microbiota-derived short-chain fatty acids in gut-bone communication and the potential of genomic selection to breed livestock with heritable metabolic resilience are explored. Ultimately, this comprehensive framework emphasizes a paradigm shift from emergency treatment to precision nutritional and genetic prophylaxis to mitigate PP across diverse ruminant species.

## Simple Summary

Parturient paresis is a severe condition in which high-yielding dairy cows and other livestock experience a sudden, dangerous drop in blood calcium levels around parturition. This health crisis causes muscle weakness, downer cow syndrome, and triggers secondary diseases, leading to major financial losses for farmers globally. This review examines the biological networks that regulate calcium balance within the animal body, specifically looking at why protective hormone systems fail to react quickly enough when milk production begins. We evaluate various modern farm management strategies used to prevent this condition, including specialized prepartum diets that prime the animal’s metabolism, digestive binding agents that enhance mineral absorption, and direct nutritional supplements like vitamins. Additionally, the paper discusses how breeding animals with natural genetic resistance can provide a permanent solution. Ultimately, shifting the focus from emergency medical treatment to precise nutritional and genetic prevention will drastically reduce animal disease, improve livestock welfare, and enhance the overall sustainability and economic security of the global dairy industry.

## 1. Introduction

Parturient paresis (PP), also known as milk fever, is a critical metabolic disorder characterized by acute, prolonged hypocalcemia during the periparturient period. Although advancements in nutritional management and therapeutic interventions have reduced incidence rates from 30% to approximately 2.8% [1,2,3,4,5] and significantly decreased mortality from 70% to near 1% [6], PP remains a substantial economic burden. This impact is particularly pronounced in emerging dairy sectors within Western Asia and Africa [2,7,8]. Beyond its primary clinical manifestations, PP serves as a predisposing factor for a cluster of secondary pathologies, including ketosis, metritis, and mastitis. These complex interactions obscure the underlying pathophysiology and continue to impede the complete control of the condition.

Current research emphasizes the relationship between milk yield, reproductive performance, and farm management [9,10,11,12], while underscoring the central role of calcium homeostasis in PP pathogenesis [1,13,14]. Despite these insights, the precise integration of these metabolic pathways has yet to be fully elucidated. It is worth noting that while modern veterinary medicine frequently addresses the broader biochemical spectrum of clinical and subclinical hypocalcemia, this review deliberately focuses on the clinical entity traditionally designated as PP. Our discussion specifically targets the acute neuromuscular progression, clinical staging, and emergency management of the severe paretic phenotype, while leaving the herd-level metabolic monitoring of standing, subclinical cohorts outside the primary scope of this work. This review provides a systematic synthesis of current knowledge regarding PP—delineating its historical context, clinical progression, risk factors, and molecular mechanisms—alongside an evaluation of modern treatment and prevention strategies. By consolidating these findings, we aim to establish a theoretical framework for developing refined management protocols and clarifying the specific metabolic pathways involved.

## 2. Review Methodology

A structured literature search was performed using the electronic databases Google Scholar, PubMed, and Web of Science to identify peer-reviewed publications published from 2010 to March 2026, with Google Scholar serving as the primary discovery engine. The search strategy utilized Boolean operators to combine core pathological terms including periparturient paresis, milk fever, and hypocalcemia with taxonomic descriptors such as dairy cows, ruminants, ewes, and goats, alongside therapeutic or preventive intervention terms such as calcium gluconate, dietary cation–anion difference, zeolite, clinoptilolite, and vitamin D.

**Figure 1:**
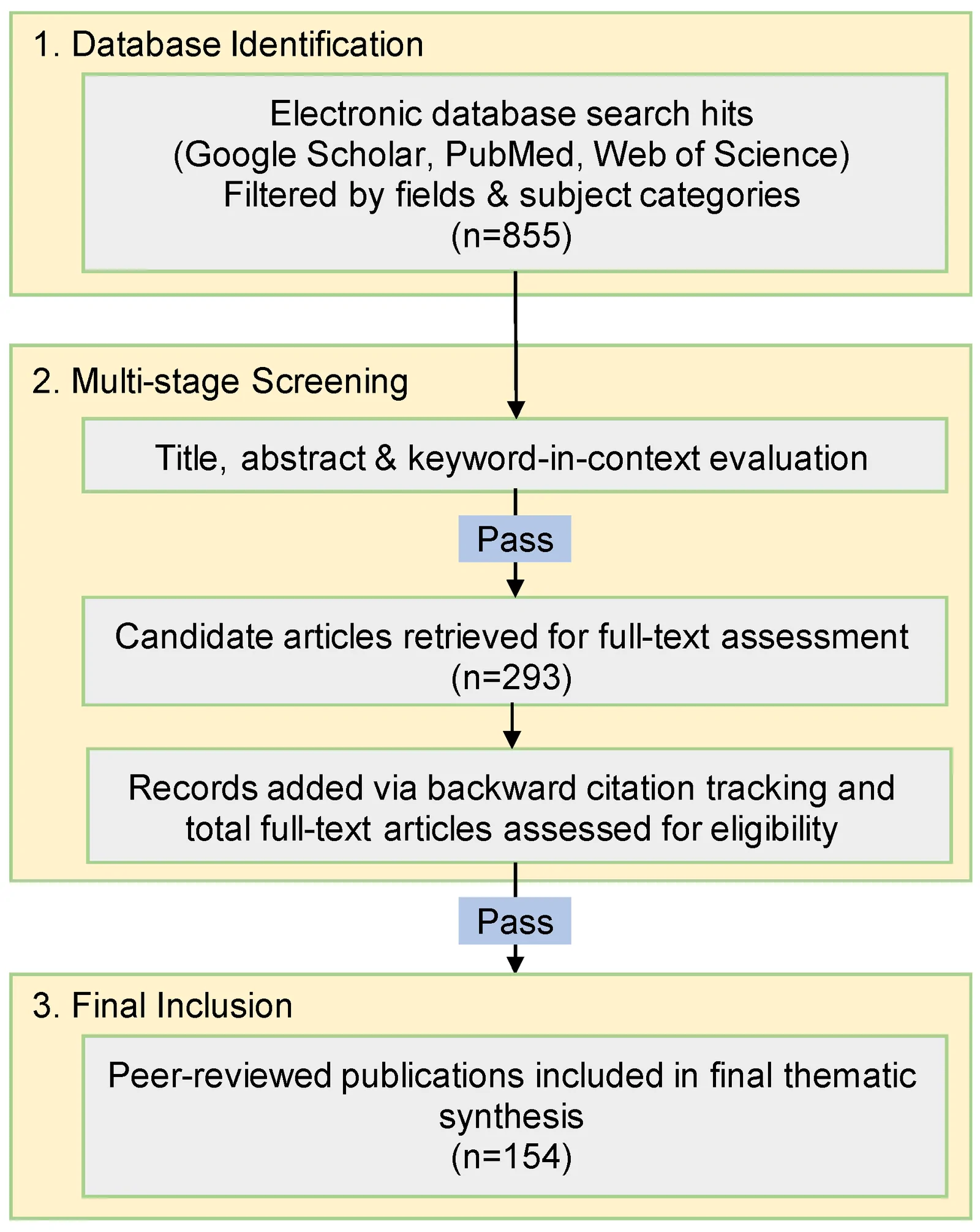
Flowchart of the multi-stage literature search and screening protocol. The diagram outlines the multi-tier selection process, starting from primary database identification (*n* = 855), progressing through title/abstract and context screening (*n* = 293), incorporating backward citation tracking, and culminating in the final set of peer-reviewed publications (*n* = 154) selected for thematic analysis.

To establish a precise and manageable scope amidst the high volume of raw search engine hits, strict pre-selection criteria were applied to the electronic database queries. Studies were considered for evaluation only if they met specific inclusion criteria, which required that they were peer-reviewed original research articles, quantitative meta-analyses, or critical reviews published in English, and that they focused explicitly on the physiological mechanisms, diagnostic biomarkers, genetic breeding values, or nutritional mitigation strategies of transition dairy ruminants. By applying these parameters—specifically restricting database search fields to titles, abstracts, and index keywords, and filtering by subject categories limited to veterinary medicine, animal science, and agriculture—the vast pool of raw search results was systematically refined to a baseline of 855 highly relevant records. To manage the high volume of search results, a rigorous multi-stage screening protocol was implemented. Candidate publications were first evaluated based on title and abstract relevance, followed by a targeted keyword-in-context analysis within the body text to confirm thematic alignment before full-text retrieval. This process narrowed the selection to 293 candidate articles. To supplement this electronic search and ensure comprehensive coverage, backward citation tracking was systematically performed on the reference lists of the retrieved articles to identify additional historically foundational or highly relevant studies that were not captured by the primary database search. Publications were excluded if they focused on non-ruminant species, lacked peer-reviewed validation, consisted solely of conference abstracts, or did not provide objective physiological parameters or quantified metabolic data. Studies published in languages other than English were also excluded. Following this combined approach, a total of 154 peer-reviewed publications were selected for final thematic analysis. Primary research articles, prospective clinical trials, and systematic reviews were included if they investigated the molecular mechanisms of calcium homeostasis, clinical progression staging, immediate parenteral or oral therapeutic protocols, or prepartum nutritional prevention strategies in cattle or small ruminants. The multi-stage screening protocol is visually summarized in Figure 1.

Due to substantial heterogeneity in experimental designs, animal breeds, dietary formulations, and reported outcome measures across the literature, a quantitative meta-analysis was not feasible. Therefore, findings were synthesized using a qualitative thematic approach, with evidence systematically categorized into physiological mechanisms, clinical staging criteria, emergency therapeutic regimens, and nutritional mitigation strategies. Because this review was based entirely on the previously published secondary literature and did not involve direct animal experimentation or sample collection, formal ethical approval was not required.

## 3. Historical Background and Nomenclature Evolution

**Figure 2:**
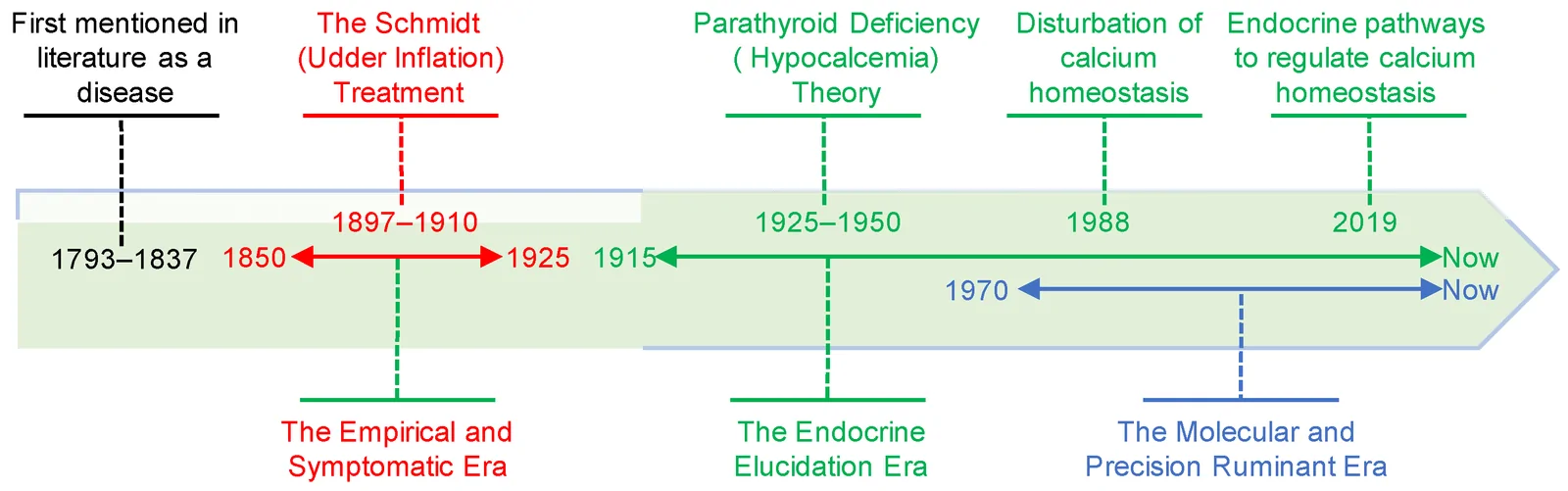
Chronological evolution of periparturient hypocalcemia research mapped across three operational eras. This timeline traces the paradigm shifts in transition cow pathology over the past century, moving from early clinical observations (Empirical Era) to endocrine regulatory mapping (Endocrine Era) and contemporary receptor-level genomics (Molecular and Precision Era).

Over the past centuries, investigations into the etiology of PP have progressively shifted from descriptive phenotypic characterization toward the precise identification of molecular biomarkers and mechanistic pathways (Figure 2).

### 3.1. The Empirical and Symptomatic Era (Pre–1925)

Early investigations into PP were strictly limited by a lack of biochemical diagnostic tools, forcing reliance on clinical observations of neuromuscular deficits and circulatory collapse. Hypotheses during this period were driven by speculative medical theories, including cerebral congestion and mammary air embolisms [15]. Although these early etiological models were subsequently invalidated, the empirical success of udder inflation therapy during this era established the historical baseline for transition cow pathology and highlighted the profound physiological impact of the mammary gland on systemic stability [16].

### 3.2. The Endocrine Elucidation Era (1925–1990)

The introduction of quantitative serological analysis shifted the research paradigm toward circulatory homeostasis. The historic debate between hypoglycemia and hypocalcemia was conclusively resolved in favor of the latter, establishing acute extracellular calcium deficiency as the primary driver of PP [17]. Subsequent research during this period successfully mapped the classical endocrine networks—principally the parathyroid hormone (PTH)–vitamin D axis—that govern calcium mobilization [18]. This biological foundation directly enabled the development of target herd-level preventative strategies, most notably the manipulation of the dietary cation–anion difference (DCAD) to induce mild metabolic acidosis [19].

### 3.3. The Molecular and Precision Ruminant Era (1970–Present)

The contemporary era has expanded the hypocalcemia paradigm to encompass target-tissue receptor mechanics and genomic predisposition. Pronounced breed-specific variations and strong antagonistic genetic correlations with milk volume confirmed a heritable architecture, with modern linear and threshold models establishing a true heritability range of 0.03 to 0.07 [20,21]. High-throughput genomic studies have transitioned the field from single-candidate gene mapping (e.g., VDR, CASR, and GC) to whole-genome prediction frameworks such as single-step GBLUP (ssGBLUP) and copy number variation (CNV) analyses [22,23]. Integrating these serum biochemical profiles with clinical symptoms remains the standard diagnostic approach, providing a multi-dimensional framework for contemporary prophylactic and therapeutic strategies.

## 4. Clinical Diagnosis and Serological Biomarkers of PP

The diagnostic landscape of PP has transitioned from a reliance on overt clinical phenotypes to the integration of precise molecular biomarkers.

### 4.1. Clinical Symptomatology

Dependent upon the severity of the systemic calcium deficit, parturient paresis typically advances through three distinct chronological stages.

The excitation period is a transient phase that typically lasts less than one hour. This stage is defined by neuromuscular hyperexcitability and mild ataxia. Affected animals exhibit localized muscle fasciculations, hind-limb shuffling, and marked hypersensitivity to external stimuli [24]. Secondary to this elevated muscular activity, animals often experience moderate tachycardia and transient hyperthermia ranging from 39.5 °C to 40.0 °C [25]. In dairy cows, the clinical onset typically occurs within 12 to 96 h postpartum, whereas the condition manifests during late gestation in ewes and does [3,26].

The sternal recumbency period ensues as serum calcium concentrations decline further, causing flaccid paralysis and deep behavioral depression. Primary clinical signs include an S-shaped cervical curvature or the permanent diversion of the head toward the flank [27]. Autonomic nervous system dysfunction leads to complete gastrointestinal stasis, which manifests clinically as ruminal bloat, constipation, and the loss of the anal reflex. Concurrently, the rectal temperature declines sharply into hypothermic levels between 25.6 °C and 27.8 °C [24].

The lateral recumbency period represents the final phase of the disorder, characterized by progressive narcosis and profound hemodynamic collapse. Peripheral reflexes are entirely abolished and the animal becomes comatose [28]. Vital signs demonstrate labored respiration and a thready, nearly imperceptible pulse with severe tachycardia exceeding 120 beats per minute. Without immediate pharmacological intervention, death from cardiac or respiratory failure occurs within hours [29].

### 4.2. Serological Biomarkers and Homeostatic Imbalance

Serum biochemical profiling serves as the gold standard for confirming PP and monitoring herd metabolic health. The clinical diagnostic criteria, threshold physiological parameters, and primary predisposing risk factors associated with the development of periparturient paresis are synthesized in Table 1.

#### 4.2.1. Macromineral Dynamics

Acute hypocalcemia constitutes the definitive hallmark of PP, although the diagnostic threshold for serum total calcium remains inconsistently defined across the clinical literature. While 2.0 mM represents the traditional benchmark for clinical diagnosis [9,30], extensive large-scale herd experiments validate 1.4 mM as the definitive threshold for severe clinical hypocalcemia [31]. The severity of mineral depletion mirrors clinical progression, with total calcium concentrations plunging to 0.5 mM during terminal stages [2].

Differential diagnosis depends on evaluating the interplay between calcium, phosphorus, and magnesium. The classic serological profile of PP involves concurrent hypophosphatemia and hypermagnesemia. Serum calcium and phosphorus maintain a consistent positive correlation, while the normal baseline serum ratio typically ranges from 1.5:1 to 2:1, and a significantly altered calcium-to-phosphorus ratio serves as a robust indicator of metabolic failure [32,33]. Conversely, magnesium concentrations frequently exhibit a negative correlation with calcium, though this dynamic is less uniform across animal populations.

Small ruminants display greater phenotypic heterogeneity during these mineral shifts. While some ovine studies mirror the classic low-calcium, low-phosphorus, and high-magnesium profile, others report divergent findings. Diseased Ossimi ewes exhibit significantly reduced concentrations of both calcium and magnesium [34], whereas Akkaraman and Awassi breeds demonstrate marked hyperphosphatemia during hypocalcemic episodes [35]. In caprine production, significant macromineral depletion is detectable as early as 14 days prior to kidding [29].

These physiological variations are driven by parathyroid hormone activity and dietary mineral intake. In response to hypocalcemia, parathyroid hormone enhances renal calcium reabsorption and bone mobilization while simultaneously promoting renal phosphaturia. This homeostatic mechanism typically induces hypophosphatemia and hypermagnesemia. However, the synergistic action of calcitriol and high dietary phosphorus intake can override these pathways, resulting in atypical elevations of serum phosphorus in certain clinical cases.

**Table 1:** Summary of Diagnostic Criteria and Influencing Factors.

Breed	Parity	Sampling Time	Calcium Measurements	Calcium Threshold	Management Systems	Reference
clinical hypocalcemia						
Holstein	1 2 3 4+	0 to 48 h after calving	spectrophotometry	2.0 mmol/L	feeding of a TMR-based diet; use of computerized herd management software	[9]
Holstein	1 2 3 4+	within 2 h after calving	spectrophotometry	2.0 mmol/L	feeding of a TMR-based diet and all cows had access to pasture	[30]
Holstein-Friesian and Jersey cows	2 3 4 5	–	–	1.4 mmol/L ^1^	–	[31]
subclinical hypocalcemia						
Holstein	1 2 3+	pre-partum and at d 1, 2, and 3 after calving	Photometric colorimetric test using 5-nitro-5′-methyl-BAPTA	2.15 mmol/L	housed in free-stall barns; fed partial mixed ration consisted of forage and concentrate	[36]
Holstein	1 2 3 4+	first 12 d in milk	quantitative colorimetric calcium test	2.1 mmol/L	feeding of a TMR-based diet	[37]
Holstein	–	atomic absorption spectrophotometry	daily from d −5 to 5 relative to parturition	1.5 to 1.85 mmol/L	–	[38]
Chinese Holstein	1 2 3 4+	within 24 h of parturition	spectrophotometry	1.65 mmol/L	All cows diets were formulated to meet the nutrient requirements recommended by the Dairy Cows Breeding Standard of China	[39]

^1^ The data is from a review article.

#### 4.2.2. Subclinical Hypocalcemia (SCH)

SCH represents a highly prevalent periparturient metabolic disorder that is etiologically linked to clinical PP. Unlike the clinical presentation, subclinical hypocalcemia manifests without overt symptoms such as recumbency or tetany. The absence of pathognomonic signs makes this condition an insidious threat to herd productivity, as its high prevalence within transition herds impairs reproductive performance and reduces subsequent lactational yield [9,36]. While both syndromes share a common origin in acute systemic calcium deficiency, the subclinical form remains undetected without laboratory screening.

Diagnostic thresholds for SCH lack global standardization and vary according to breed and herd management systems. Total serum calcium concentrations of 2.15 mM or lower are frequently used to define the condition [36,37]. Severe manifestations are categorized within a narrower range of 1.5 to 1.85 mM [38]. Other established diagnostic parameters identify subclinical values spanning from 1.4 to 2.0 mM [31]. In specific populations such as Chinese Holstein cows, affected individuals demonstrate a mean total calcium concentration of 1.65 mM, which stands in contrast to the 2.26 mM baseline observed in healthy cohorts [39]. This systemic calcium deficit does not occur in isolation but rather correlates with broader metabolic dysregulation. Affected cows frequently present with altered serum magnesium and phosphorus profiles alongside elevated circulating concentrations of non-esterified fatty acids and beta-hydroxybutyrate. This biochemical overlap demonstrates that subclinical hypocalcemia is linked with negative energy balance and impaired hepatic lipid metabolism, which collectively compounds the physiological challenges of the transition period.

## 5. Molecular Mechanisms Underlying Calcium Dyshomeostasis and Feedback Failure in PP

**Figure 3:**
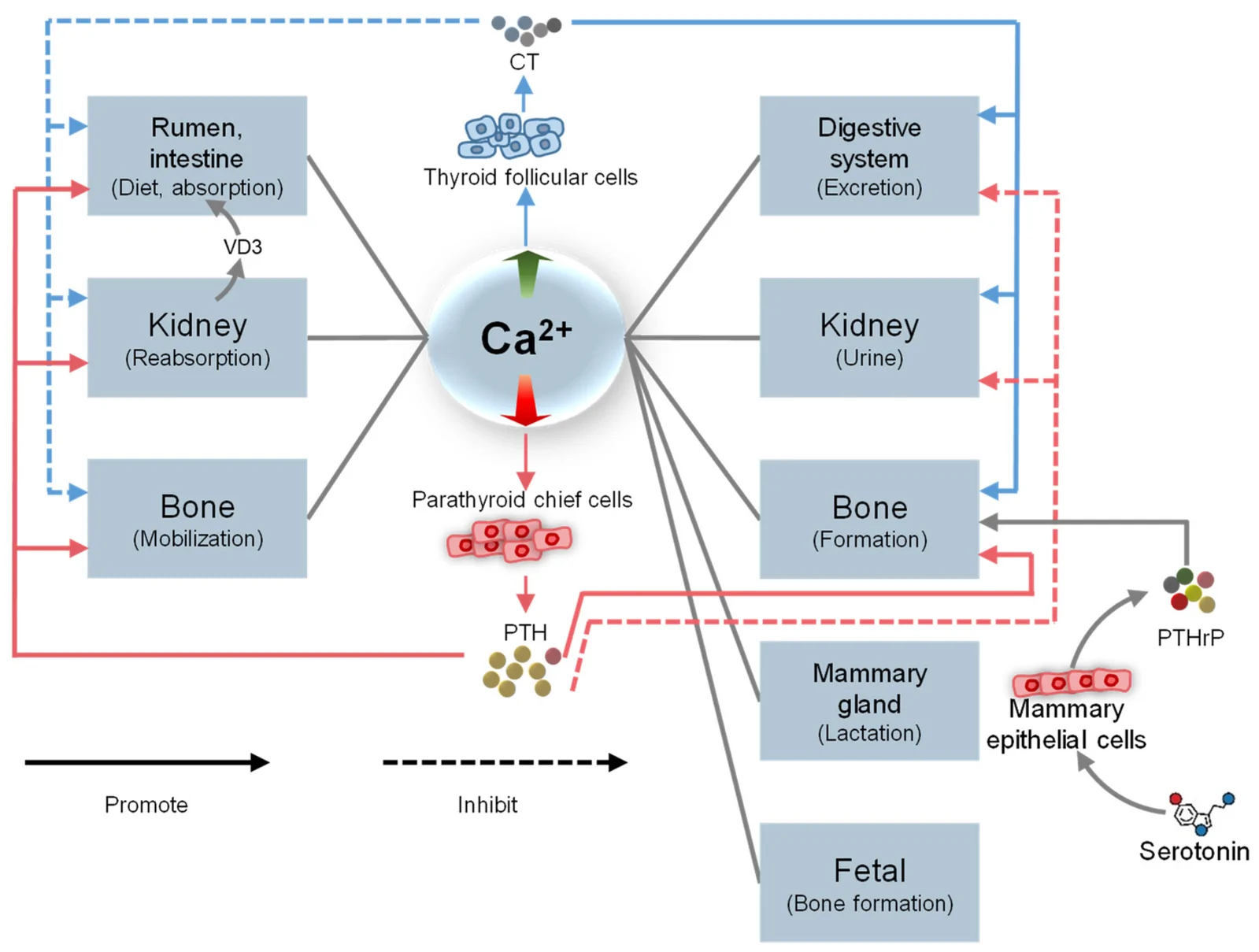
Inputs and outputs to the plasma calcium pool.

In mammals, serum calcium homeostasis is maintained through a precisely regulated equilibrium involving intestinal absorption, renal reabsorption, and skeletal mobilization. However, during the periparturient period, the onset of colostrogenesis and fetal skeletal mineralization imposes a significant physiological demand for calcium, which frequently exceeds the homeostatic regulatory capacity (Figure 3). This acute transition initiates a negative calcium balance, as the rate of calcium efflux from the extracellular fluid exceeds the rate of replenishment from endogenous sources, thereby compromising systemic calcium homeostasis. Clinical data across ruminant species consistently demonstrate a marked decline in serum calcium in animals diagnosed with PP. Specifically, total calcium levels in affected dairy cows often decrease to ≤1.4 mM (versus ≥2.0 mM in healthy counterparts), while levels in affected ewes decrease to ≤1.0 mM (versus ≥1.5 mM in healthy sheep). The definitive causal relationship between acute hypocalcemia and the clinical presentation of recumbency has been established through Na2EDTA infusion models, which verify that the rapid sequestration of ionized calcium is sufficient to induce the PP phenotype [40]. These findings collectively underscore that the clinical manifestation of PP represents a failure of the homeostatic feedback system to synchronize calcium mobilization with the sudden surge in output requirements.

**Figure 4:**
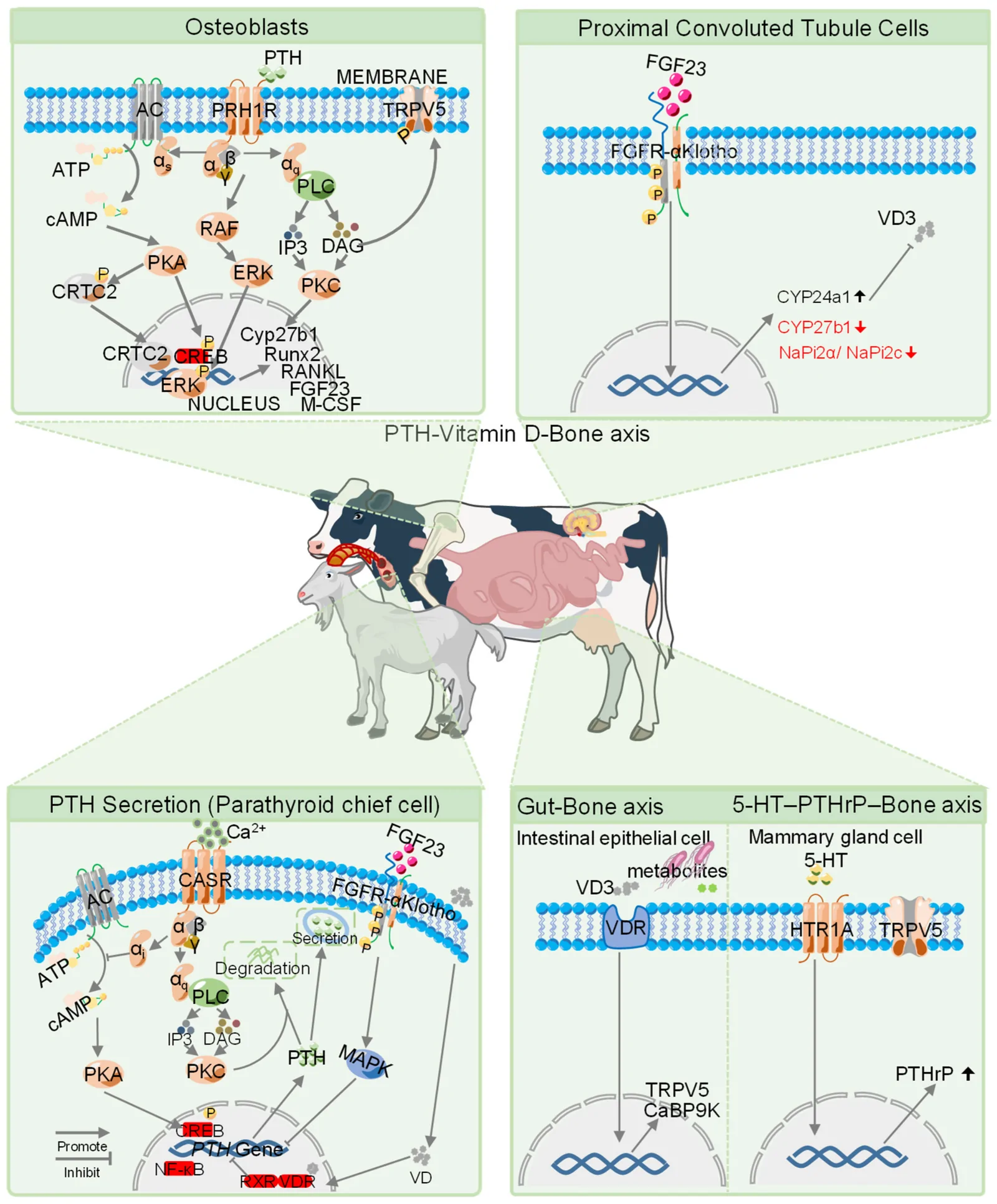
Intracellular signaling pathways and endocrine feedback networks governing periparturient calcium homeostasis. The parathyroid chief cell model illustrates parathyroid hormone synthesis, secretion, and degradation pathways controlled by calcium-sensing receptor activity and fibroblast growth factor 23 signaling. The osteoblast and proximal convoluted tubule models depict parathyroid hormone receptor 1 activation triggering downstream cyclic adenosine monophosphate and phospholipase C cascades that regulate bone remodeling factors alongside renal vitamin D activation and phosphate cotransporter expression. The intestinal epithelial and mammary gland models demonstrate active transcellular calcium transport regulation via the vitamin D receptor alongside serotonin-driven synthesis of parathyroid hormone-related protein via the serotonin receptor 1A. Upward arrows (↑) indicate an upregulation, while downward arrows (↓) indicate a downregulation.

Calcium homeostasis in ruminants is a tightly orchestrated process governed by a suite of humoral factors, including parathyroid hormone (PTH), 1,25(OH)_2_D_3_, fibroblast growth factor 23 (FGF23), 5-hydroxytryptamine (5-HT), PRL, and calcitonin (CT). Among them, PTH–1,25(OH)_2_D_3_–bone axis is the classical regulating pathway, gut–bone axis and 5-HT–parathyroid hormone-related protein (PTHrP)–bone axis are new pathways. The synergistic or antagonistic modulation of these pathways determines the animal’s ability to maintain systemic calcium levels amidst the metabolic shifts in the periparturient period (Figure 4).

### 5.1. Classical Regulatory Pathways of Calcium Homeostasis

Systemic calcium homeostatic stability during the periparturient transition relies on the rapid, coordinated interplay between counter-regulatory endocrine axes. This baseline physiological framework is governed primarily by the cooperative actions of the parathyroid hormone–vitamin D axis and the counter-active stabilizing dynamics of calcitonin.

#### 5.1.1. The Parathyroid Hormone–Vitamin D Axis

PTH secretion is primarily regulated by the calcium-sensing receptor (CaSR) on parathyroid chief cells. Hypocalcemia diminishes CaSR-mediated inhibition, activating a G-protein–PLC–IP_3_/DAG pathway that stimulates endoplasmic reticulum calcium release and protein kinase C (PKC) to drive PTH exocytosis [41,42]. Beyond acute secretion, long-term PTH availability is regulated by VDR/RXR and FGFR1/Klotho-mediated transcriptional repression [43,44,45], alongside post-transcriptional mRNA stability modulated by AUF1 and KSRP [41,42,46,47]. Circulating PTH binds to target tissue parathyroid hormone 1 receptor (PTH1R) to trigger the AC/cAMP/PKA pathway [48]. In the kidneys, this cascade upregulates cytochrome P450 27B1 (CYP27B1) expression to convert 25(OH)D_3_ into active 1,25(OH)_2_D_3_, a process amplified by the PTH–SIK–CRTC axis where salt-inducible kinase (SIK) inhibition permits CRTC2 binding to *Cyp27b1* enhancers [49,50] (Figure 4).

Acute hypocalcemia elicits a robust compensatory parathyroid response across affected herds, though reported literature values diverge numerically due to shifts in assay specificity. Early endocrinological investigations utilizing polyclonal radioimmunoassays reported that paretic dairy cows mount a total immunoreactive PTH surge ranging from 4 to 9 ng/mL [18,51]. This historical methodology quantified both the active hormone and its abundant circulating degradation fragments, yielding highly elevated mass concentrations. In contrast, modern clinical evaluations focus exclusively on the biologically active intact parathyroid hormone fraction using monoclonal sandwich assays. Under these refined standards, hypocalcemic cows exhibit a parallel compensatory rise with intact PTH concentrations stabilizing between 6.84 and 13.35 pmol/L [52,53]. Rather than indicating a systemic feedback failure, this numerical gap reflects the technical transition from total fragment profiling to intact hormone quantification, with both eras confirming a vigorous endocrine defense during acute calcium drainage.

**Figure 5:**
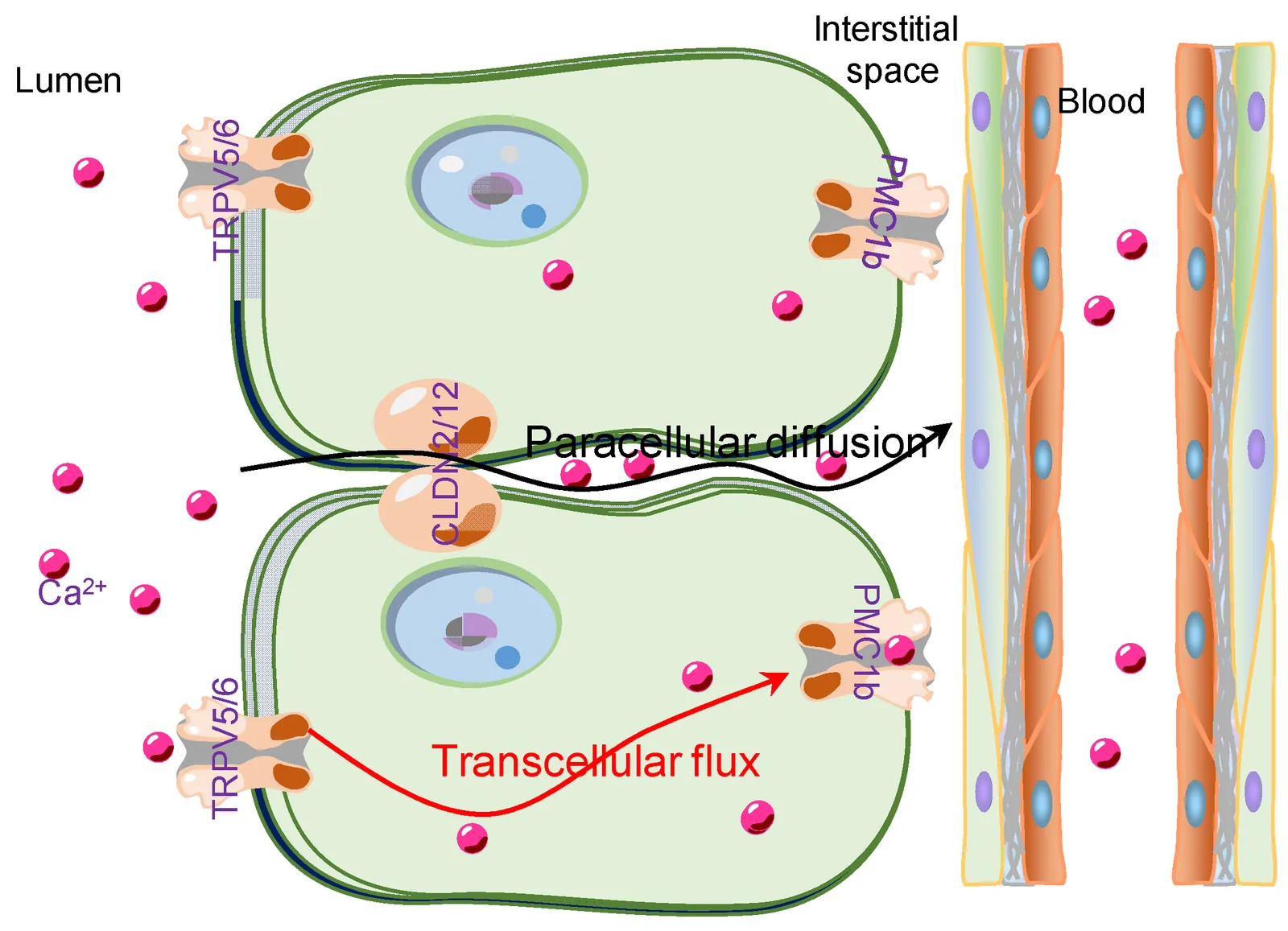
The pathway of calcium absorption, resorption and transport.

Downstream of this endocrine output, epithelial calcium transport utilizes passive paracellular and active transcellular pathways. Driven by electrochemical and osmotic gradients, the paracellular pathway operates primarily in the intestinal epithelia and renal proximal tubules via tight junction proteins Claudin-2 (CLDN2) and Claudin-12 (CLDN12) [54,55,56,57]. Conversely, the transcellular pathway dominates during dietary restriction or high physiological demand. Calcium enters apical channels—transient receptor potential channel subfamily v member 6 (TRPV6) in the intestine and transient receptor potential channel subfamily v member 5 (TRPV5) in distal renal tubules—undergoes intracellular shuttling via Calbindin-D9K to prevent cytotoxicity, and is extruded basolaterally into the bloodstream by plasma membrane calcium ATPase (PMCA1b) [55,56,57] (Figure 5) [58,59,60]. This active process depends on 1,25(OH)_2_D_3_ binding to the intracellular vitamin D receptor (VDR). The ligand-bound VDR heterodimerizes with the retinoid X receptor (RXR), translocates to the nucleus, and binds vitamin D response elements (VDRE) to transactivate and upregulate TRPV6, Calbindin-D9k, and PMCA1b expression [55,61].

#### 5.1.2. Calcitonin Counter-Regulatory Dynamics

Calcitonin serves as a physiological antagonist to parathyroid hormone, secreted by thyroid parafollicular cells during hypercalcemia to suppress bone mobilization and promote renal mineral excretion [62,63]. Upon binding its G-protein-coupled receptor on osteoclasts, calcitonin initiates the dephosphorylation of proline-rich tyrosine kinase 2 and the concurrent phosphorylation of focal adhesion kinase [64]. This signaling shift alters cytoskeletal structure, provoking osteoclast retraction and suppressing migratory capacity to halt bone resorption [65]. Knockout models suggest a dual role where calcitonin limits pathological skeletal mineral loss during high demands [66], yet may also modulate bone formation through the OPG/RANKL system or osteoclast-derived Wnt10b expression [67]. In transition dairy cows, calcitonin dynamics display a pathological paradox [39]. While certain cohorts exhibit stable concentrations, severely hypocalcemic cows demonstrate a 2.85-fold increase in circulating calcitonin at calving [38]. This inappropriate elevation of a potent osteoclast inhibitor at the exact onset of lactation stalls bone mobilization, directly impeding the homeostatic compensation required to resolve the acute mammary calcium deficit and accelerating the transition from subclinical hypocalcemia to clinical PP.

### 5.2. Pathophysiological Mechanisms of Feedback Failure

While classical endocrine pathways provide a robust framework for systemic calcium balance, the acute transition into lactation frequently introduces severe physiological disruptions that cause these feedback loops to fail. This regulatory breakdown does not stem from a lack of hormone production, but rather from systemic imbalances that manifest as rate mobilization mismatches, secondary messenger blockade, and downstream tissue receptor resistance.

#### 5.2.1. Kinetic Dyssynchrony and Bone Mobilization Lags

The susceptibility of dairy ruminants to PP is determined by a rate-differential mismatch between mammary calcium clearance and homeostatic recruitment velocity rather than absolute milk volume. Intensified genetic selection for high milk yields directly correlates with this metabolic failure, where a 1500 kg lactation yield above breed average elevates the odds of PP by 2.39-fold, and risk rises sharply in herds yielding over 9150 L annually [68,69]. This vulnerability centers on the mammary calcium drain phenomenon during early colostrogenesis. Specifically, population data for multiparous cows reveal a complex physiological paradox where individuals with a serum total calcium concentration below 2.1 mmol/L exhibit a statistical tendency to produce 0.80 kg per day more milk during early lactation compared to their normocalcemic herd mates [9], which functions as an epidemiological risk indicator rather than a direct causal driver of performance outcomes. This rapid, active translocation of ionized calcium into the alveolar lumen against a steep electrochemical gradient creates an acute systemic deficit that outpaces immediate homeostatic capacity across all high-yielding ruminants.

Gastrointestinal replenishment fails to match this instantaneous efflux due to the inherent latency of epithelial transport upregulation. During acute depletion, the physical efflux rate regularly exceeds the maximal transport threshold of passive paracellular and active transcellular pathways. Because the genomic transactivation, transcription, and translation of these epithelial transport proteins, like TRPV6 and PMCA1b, require an induction period spanning several hours to days, intestinal absorption remains temporarily fixed at prepartum baseline levels, demonstrating that early homeostatic failure stems from upstream endocrine latency rather than structural transporter defects.

This supply-demand deficit is compounded by the biological lag in skeletal remodeling under the dual burden of fetal mineralization and lactogenesis. Normocalcemia maintains active WNT/β-catenin signaling to drive osteoblastic bone deposition [70]. Conversely, acute systemic deficits require an immediate metabolic pivot to skeletal resorption mediated through the nuclear factor kappa-B ligand (RANKL)/nuclear factor kappa-B (RANK)–Osteoprotegerin (OPG) axis, where receptor activator of nuclear factor kappa-B ligand stimulates osteoclast precursor maturation and mineral mobilization [71,72,73,74] (Figure 4). However, the cellular transition from WNT-driven deposition to mature RANKL-mediated resorption requires a distinct temporal induction phase. Consequently, PP is fundamentally a disease of kinetic dyssynchrony, defined by the temporal misalignment between the operational velocity of the endocrine-skeletal feedback machinery and the acute acceleration of peripheral calcium efflux.

#### 5.2.2. Receptor Downregulation and Target-Tissue Resistance

While this kinetic mismatch creates an inevitable post-partum deficit, the severity of the metabolic drop depends heavily on downstream target-tissue responsiveness. Although the parathyroid glands attempt a vigorous compensatory response during acute calcium drainage, the downstream physiological efficacy of this axis can be severely blunted by concurrent metabolic and dietary interference. This endocrine failure is frequently exacerbated by hypomagnesemia. Insufficient extracellular magnesium deprives adenylate cyclase of its obligate magnesium cofactor, thereby restricting intracellular cAMP synthesis within target tissues and delaying postpartum calcium recovery [75]. Concurrently, DCAD dictates receptor compliance. High positive DCAD diets induce mild metabolic alkalosis, increasing PP susceptibility [76]. In contrast, reducing DCAD to neutral or negative values ranging from 0 to −100 mEq/kg DM decreases clinical incidence of PP by up to 87% [77,78]. The physiological efficacy of a negative DCAD resides in its capacity to induce compensated metabolic acidosis, which optimizes the stereochemical configuration of PTH1R on bone and kidney tissues, thereby restoring receptor sensitivity to accelerate mineral mobilization and renal calcitriol synthesis.

Beyond these ionic interferences, a kinetic lag between hypocalcemia onset and peak endogenous 1,25(OH)_2_D_3_ synthesis further delays homeostatic adjustment [79]. This systemic vulnerability is severely exacerbated by an age-dependent reduction in VDR density within intestinal and osseous target tissues, precipitating peripheral receptor resistance where elevated circulating calcitriol fails to elicit sufficient target-organ responses. This molecular resistance accounts for the epidemiological divergence between parities; clinical PP is rare in primiparous heifers, whereas cows in their fifth or sixth lactation exhibit a 12- to 31-fold higher probability of developing PP than those in their second lactation [31,80]. This senescent vulnerability is driven by concurrently declined bone mineral mobilization, diminished enterocyte transport capacity, and attenuated renal CYP27B1 activation (Figure 4).

Unlike the bovine model, both primiparous and older small ruminants are susceptible to PP due to the physiological strain of early mating in younger animals and lower baseline digestive or absorptive capacities in older cohorts, with caprine herds over three years of age reaching a 60% incidence rate [29]. Furthermore, breed-specific variations demonstrate a genetic link to receptor expression. Jersey and Guernsey cattle carry up to a five-fold higher relative risk than Holsteins, a predisposition mapped to a lower constitutional density of intestinal VDRs paired with a high ratio of milk production relative to metabolic body weight [4,31,68]. Jersey–Holstein crosses show intermediate susceptibility [31], while temperate breeds exhibit a consistently higher predisposition than tropical Zebu or adapted local breeds [81]. Thus, while elevated milk yield drives the baseline metabolic load, heritable genomic architectures govern these breed-specific variations in receptor sensitivity and homeostatic efficiency.

#### 5.2.3. Fibroblast Growth Factor 23 as a Pathophysiological Brake

Excessive prepartum dietary phosphorus intake exceeding 80 g per day constitutes a major nutritional driver of PP, operating through the premature induction of FGF23 [81]. Secreted by osteocytes and osteoblasts in response to elevated systemic phosphorus or calcitriol, this bone-derived osteokine functions as a physiological antagonist to calcium mobilization. However, the causal relationship between FGF23 dynamics and PP development in periparturient ruminants requires cautious interpretation due to clear limitations in the existing evidence base. While the epidemiological association between high prepartum phosphorus and postpartum hypocalcemia is well documented in dairy cattle, the downstream intracellular signaling cascade is largely inferred from non-ruminant models.

In the human renal proximal tubules, circulating FGF23 binds to the FGFR1-alpha-Klotho receptor complex, utilizing alpha-Klotho as an obligate co-receptor to ensure tissue specificity. This signaling cascade induces rapid phosphaturia by downregulating the expression of apical sodium-phosphate cotransporters, specifically NaPi-IIa and NaPi-IIc, to increase urinary phosphorus excretion [82]. Human medical studies also demonstrate that the renal FGFR1-Klotho complex directly represses the transcription of CYP27B1, the rate-limiting enzyme responsible for activating 25(OH)D_3_ into 1,25(OH)_2_D_3_; simultaneously, it upregulates the transcription of CYP24A1, which accelerates the catabolism of active calcitriol into inactive metabolites [79] (Figure 4). This reciprocal enzymatic regulation severely restricts the circulating calcitriol pool required for active enterocyte calcium transport. Furthermore, FGF23 participates in a long-loop negative feedback system by binding to FGFR1-Klotho complexes on parathyroid chief cells, directly suppressing the transcription and secretion of PTH [83].

Direct empirical validation of this precise reciprocal enzymatic regulation and its long-loop negative feedback remains highly scarce in transition livestock. Most available ruminant investigations rely on observational dietary trials demonstrating that high prepartum phosphorus depresses postpartum calcium levels, rather than on direct measurements of active circulating FGF23 proteins during the transition period. This empirical gap is further widened by a lack of validated, ruminant-specific commercial assays capable of distinguishing active intact FGF23 from its inactive degradation fragments during acute metabolic crises. Without longitudinal endocrine profiles tracking intact FGF23 concentrations across the periparturient phase, the role of this osteokine as a definitive causal mechanism in ruminant parturient paresis remains a hypothesis awaiting rigorous validation. When high-producing ruminants experience the sudden postpartum calcium drain of colostrogenesis, this prepartum FGF23-mediated inhibition leaves the renal and parathyroid machinery unresponsive, precipitating a failed calcemic response and the onset of PP. Consequently, maintaining an optimal dietary calcium-to-phosphorus ratio near 2.3 to 1 is critical to avoid triggering this early endocrine disruption and preventing secondary suppression of the homeostatic response.

### 5.3. Emerging Regulatory Networks and Systemic Inputs

Beyond the classical endocrine axes and their localized receptor mechanics, systemic calcium homeostasis is heavily modulated by peripheral signaling networks and whole-body energy states. This final section explores how the mammary serotonin-prolactin axis, maternal nutritional energetics, and microbiota-derived metabolites integrate to influence periparturient calcium flux, while critically evaluating the translational validity of these emerging pathways from monogastric models to ruminant physiology.

#### 5.3.1. The Mammary Serotonin–Prolactin Axis

Peripheral serotonin synthesized via tryptophan hydroxylase 1 (TPH1) within the intestines, bone, and mammary glands serves as an integral systemic orchestrator of calcium homeostasis during lactation [84,85]. In periparturient dairy cows, circulating serotonin concentrations demonstrate a significant positive correlation (*r* = 0.51) with systemic calcium levels, whereas diminished serotonin levels map to impaired osseous mobilization and increased PP susceptibility [86,87]. Mechanistically, peripheral serotonin acts through the mammary-PTHrP–bone axis, where the mammary gland functions as a metabolic sensor that secretes PTHrP into the circulation to stimulate bone mineral mobilization. Experimental models show that knocking down TPH1 in rodent mammary epithelial cells suppresses both serotonin and PTHrP expression [88,89], while supplementation with 5-hydroxytryptophan restores these transcripts, activates osteoclast differentiation via receptor activator of nuclear factor kappa-B ligand pathways, and enhances bone resorption [88]. Whole-transcriptome sequencing indicates that serotonin coordinates this mouse mammary transport architecture by modulating the MAPK/ERK signaling cascades alongside upregulation of apical calcium-sensing receptors and basolateral plasma membrane calcium ATPase 2 pumps [89]. In small ruminants, recent in vitro evidence utilizing goat mammary epithelial cells has successfully validated the localized functionality of this regulatory network. Experimental manipulation of serotonin synthesis in these caprine cells demonstrates that 5-hydroxytryptophan increases PTHrP expression in a dose-dependent manner, whereas siRNA-mediated knockdown of TPH1 significantly decreases PTHrP expression. Furthermore, serotonin treatment upregulates the mRNA abundance of the calcium-sensing receptor in a dose-dependent manner while concurrently decreasing the expression of plasma membrane calcium ATPase 1 [85]. Taken together, these cellular findings indicate that enhancing serotonin biosynthesis represents a viable therapeutic target for preventing hypocalcemia.

PRL undergoes a dramatic elevation exceeding tenfold during the periparturient transition to support the onset of lactation [90]. Beyond its established lactogenic functions, PRL acts as a systemic regulator of calcium flux by priming the gastrointestinal tract and skeletal tissue for elevated mineral demands. In rodent models, elevated circulating PRL enhances transcellular enterocyte absorption by upregulating apical TRPV6 channels and basolateral PMCA1 pumps [49,91]. Concurrently, PRL modulates skeletal remodeling by suppressing the transcription of Runt-related transcription factor 2 [92], which dampens osteoblast differentiation and shifts the balance toward net mineral mobilization into the systemic pool.

In contrast, direct empirical validation of these mechanisms is lacking in cattle, sheep, and goats. These discrepancies are largely driven by species-specific variations in prolactin receptor structure and signaling cascades. Structurally, the mature 557 amino acid ruminant long-form receptor lacks the terminal tyrosine residue equivalent to Y580 in rodents, inherently altering downstream STAT5-mediated transcription. Additionally, alternative splicing mechanisms differ fundamentally. While splicing strategies are conserved across humans, rodents, and pigs, the short-form receptor in bovine, ovine, and caprine species is uniquely generated by splicing from exon 9 to a 39 base pair insert upstream of exon 10. This ruminant-specific event yields a truncated protein lacking the critical Box 2 motif [93]. Because these structural differences alter receptor dimerization kinetics and potentially exert dominant-negative effects, the rodent STAT5-mediated upregulation of calcium transport proteins cannot be assumed in transition ruminants. Consequently, the function of prolactin as an active hypercalcemic agent remains an unverified hypothesis in cattle, sheep, and goats due to these profound signaling and structural variations.

#### 5.3.2. Nutritional Energetics and Voluntary Feed Intake Effects

Extended dry period durations and excessive maternal adiposity represent significant management-driven determinants that predispose transition ruminants to PP through systemic metabolic quiescence and postpartum anorexia. Dairy cows experiencing a non-lactating interval exceeding 60 days are approximately three times more likely to develop clinical PP compared to cohorts with a dry period under 30 days [68,94]. This elevated risk is associated with a prolonged state of metabolic quiescence within the calcium-regulating axis or excessive fat accumulation during elongated non-lactating intervals. Consequently, overconditioning at parturition, defined by a body condition score (BCS) of 3.5 or higher on a 5-point scale, serves as a robust predictor of homeostatic failure. Animals presenting with a BCS of 4.0 or greater exhibit a 3.3- to 4-fold higher risk of clinical PP than those maintained at optimal condition [53,95,96]. The primary underlying mechanism driving this susceptibility is the profound anorectic effect associated with excessive adiposity. Overconditioned individuals undergo a severe depression in postpartum dry matter intake, which critically restricts dietary calcium ingestion during the sudden onset of lactogenesis [31]. This nutritional deficit precipitates a cascade of secondary metabolic disorders, including ketosis, dystocia, and retained fetal membranes, ultimately compounding the systemic imbalance between high mammary calcium export and impaired mineral intake [97].

#### 5.3.3. Gut Microbiota and Metabolite Signaling Pathways

This nutritional and dry matter intake depression further compromises systemic calcium homeostatic capacity by disrupting gut-bone communication pathways mediated by the gastrointestinal microbiota [98,99,100,101,102]. In laboratory rodent models, microbiota-derived short-chain fatty acids, specifically butyrate, function as critical signaling molecules that modulate PTH-induced bone remodeling [103]. Anorexia or dysbiosis-induced depletion of luminal butyrate impairs both the anabolic and catabolic tissue responses to PTH, whereas adequate butyrate availability is required to maintain these homeostatic functions. Under conditions of optimal butyrate and endocrine signaling in mice, the intracellular cascade within osteoblasts involves the protein kinase A-induced phosphorylation of cAMP response element-binding protein (CREB), which activates receptor activator of RANKL expression [104,105]. This process is further augmented by matrix metalloproteinase 14 (MMP14) activity, which facilitates osteoclast differentiation and bone mineral mobilization to restore circulating calcium levels [106] (Figure 4). In transition ruminants, the direct application of this gut–bone axis remains entirely hypothetical. Current ruminant data are restricted to broad association studies linking postpartum anorexia and altered ruminal volatile fatty acid profiles with systemic hypocalcemia. Direct empirical evidence confirming that ruminal or intestinal butyrate fluctuations cross the epithelial barrier to actively dictate osteoblastic signaling in transition cows has not yet been established.

## 6. Treatment and Prevention Strategies

The management of PP has transitioned from reactive clinical intervention to proactive, physiology-based metabolic regulation aimed at maintaining systemic calcium flux stability. Modern evidence-based strategies integrate molecular homeostatic pathways with herd management data to optimize the physiological transition from late gestation to colostrogenesis. To enhance readability and provide a practical synthesis of these diverse approaches, the mechanisms of action, stages of application, therapeutic advantages, inherent limitations, and associated levels of evidence for both conventional and emerging interventions—including parenteral therapies, nutritional modulations, and genomic selections—are comprehensively contrasted in Table 2.

### 6.1. Parenteral and Oral Calcium Administration

Intravenous administration of calcium salts provides an immediate systemic bolus to restore neuromuscular function in clinically recumbent animals. Commercial formulations typically utilize calcium gluconate supplemented with magnesium chloride and boric acid to stabilize the solution and address concurrent mineral deficits at a standard dosage of approximately 20 mg/kg of body weight [108]. However, pharmacodynamic evaluations indicate that this parenteral infusion triggers a supra-physiological spike in plasma calcium that suppresses parathyroid hormone secretion and induces an acute calcitonin surge [75,108]. Prophylactic IV administration in normocalcemic or subclinical hypocalcemic animals is therefore highly contraindicated, as this homeostatic disruption precipitates severe secondary rebound hypocalcemia. For recumbent cows, immediate IV intervention is mandatory to prevent acute cardiopulmonary collapse; however, to mitigate the subsequent endocrine lag phase, the initial bolus should be combined with follow-up oral calcium supplementation to successfully maintain plasma calcium levels above 8.0 mg/dL [107,108].

Conversely, oral calcium supplementation serves as a prophylactic measure that leverages passive paracellular diffusion across the ruminal and intestinal epithelia to provide a gradual, sustained elevation of serum calcium without triggering hormonal feedback suppression [109,134]. Calcium chloride offers high solubility and rapid absorption, reducing the incidence of PP from 36% to 23% [110,111], although excessive or repeated doses can cause gastrointestinal epithelial irritation and metabolic acidosis [135]. Non-caustic alternatives include calcium propionate, which simultaneously supplies gluconeogenic precursors to support energy balance, and calcium carbonate, which provides a moderate duration of effect up to 24 h [1,75]. Standard oral protocols require an initial dose of 50 to 125 g of elemental calcium at the first signs of calving followed by a secondary dose 24 h later [1,75].

While parenteral calcium intervention is a universal emergency protocol across domestic ruminants, therapeutic strategies must be precision-adjusted for ewes and does due to distinct physiological timelines and body masses. Unlike dairy cattle, where clinical hypocalcemia is predominantly a postpartum phenomenon driven by colostrogenesis, the condition in small ruminants manifests primarily during late gestation, precipitated by the massive skeletal calcification demands of multiple fetuses. Consequently, therapeutic intervention requires a downscaled dosage, typically involving the subcutaneous or slow intravenous administration of 50 to 100 mL of a 20% calcium gluconate solution per animal [112]. Care must be exercised during IV administration in small ruminants to avoid fatal cardiac arrhythmias, and concurrent treatment for pregnancy toxemia (ketosis) is frequently required, as these two metabolic crises commonly coexist and exacerbate one another in multi-bearing ewes and does [136,137].

### 6.2. Prepartum Calcium Restriction and Gastrointestinal Binding Agents

Prepartum dietary calcium restriction aims to proactively stimulate parathyroid gland activity and enhance bone mineral mobilization prior to parturition. While some investigations indicate that low-calcium diets under 20 g per day reduce PP incidence [138,139], achieving this threshold is often unfeasible in commercial operations due to the naturally high mineral content of premium forages [140,141]. In addition, a late-gestation cow requires approximately 14 to 22 g/day of calcium (NRC, 2001) [142], the abrupt transition to a high-calcium lactation diet immediately after calving requires precise management to avoid metabolic shock.

To overcome these formulation constraints, nutritional strategies utilize synthetic silicate binders, such as Zeolite A, to reduce gastrointestinal calcium bioavailability. Operating as a chelating agent within the digestive tract, Zeolite A binds dietary calcium and phosphorus to facilitate fecal excretion, thereby inducing a functional hypocalcemia that activates the endogenous parathyroid hormone-calcitriol axis without absolute dietary mineral removal. Prepartum supplementation with 0.5 to 1.0 kg per day of Zeolite A during the final two to three weeks of gestation can reduce clinical incidence from 37.5% to 0% [117,118,119]. Furthermore, lower inclusion rates of 150 to 300 g per day effectively mitigate subclinical hypocalcemia, improving post-parturient plasma calcium stability and subsequent milk yield [120,121].

### 6.3. Dietary Cation–Anion Difference and Acid–Base Modulation

DCAD manipulation stands as a highly effective nutritional intervention that alters the systemic acid–base status of the transition cow to optimize tissue sensitivity to parathyroid hormone. While standard guidelines require precise baseline daily mineral intakes approximately 1.2 g of Na, 3.5–4.0 g of Mg, 8.5–10 g of Ca, 4 g of P, and 4 g of S), the primary regulatory mechanism involves the control of potassium and chlorine concentrations. Effective prevention programs maintain potassium near the baseline requirement of 10 g/kg of dry matter while ensuring the chlorine concentration remains approximately 5 g/kg below the potassium concentration to achieve the target ionic balance [75].

Meta-analytical data covering 1571 cows confirm that reducing DCAD below a differential threshold of 205 mEq/kg of dry matter significantly mitigates clinical hypocalcemia, yielding a risk ratio of 0.60 [78,143]. However, pursuing aggressive negative targets between −100 and −150 mEq/kg of dry matter can induce uncompensated metabolic acidosis and reduce dry matter intake due to the inherent unpalatability of anionic salts, potentially exacerbating postpartum negative energy balance. Severe prepartum acidification may also negatively correlate with circulating vitamin D3 concentrations, compromising long-term homeostatic capacity [113]. Consequently, current industry formulations favor neutral targets between 0 and +30 mEq/kg of dry matter or moderate negative targets near −53 mEq/kg of dry matter to enhance postpartum plasma calcium without suppressing appetite [77,114]. The physiological efficacy of these formulations is routinely verified through the systematic monitoring of urine pH.

In small ruminant management, the application of DCAD manipulation and alternative mineral-binding strategies requires a distinct physiological approach compared to dairy cattle paradigms. Recent evidence demonstrates that feeding negative DCAD diets or anionic salts to twin-bearing ewes during late gestation or peri-parturient dairy sheep can assist in mineral homeostasis [115]; however, its implementation warrants extreme caution, as alterations in DCAD levels significantly affect dry matter intake, nutrient digestibility, rumen fermentation characteristics, and the structure of the rumen microbiota [116]. To mitigate the palatability and intake risks associated with aggressive anionic salt feeding in late-pregnant ewes, alternative nutritional technologies are increasingly utilized. Specifically, dietary supplementation with natural or nano-sized zeolites serves as an innovative mechanism to manipulate phosphorus, calcium, and magnesium utilization in growing lambs and dairy goats [144,145]. These mineral supplements optimize ruminal fermentation, stabilize blood metabolites, and support milk yield without triggering systemic acid–base distress, offering a safer alternative or complementary strategy to traditional DCAD acidification for ensuring transition health in small ruminants.

### 6.4. Exogenous Vitamin D Supplementation Modulating the Parathyroid Hormone–Vitamin D Axis

Exogenous vitamin D supplementation represents a fundamental strategy for preventing PP due to its regulatory role within the parathyroid hormone–vitamin D axis. The clinical efficacy of standard cholecalciferol depends heavily on its molecular form, dosage, and administration timing relative to parturition. While historical guidelines recommended 10,000 to 12,000 IU per day, contemporary production standards advocate for higher prophylactic dosages ranging from 30,000 to 50,000 IU per day to support elevated metabolic demands [122,123,124]. Targeted prepartum oral supplementation of 3 mg per day initiated three to five days prior to calving significantly bolsters periparturient serum calcium concentrations [126]. However, a physiological saturation threshold exists where escalating crude VD3 supplementation to extreme ranges between 60,000 and 120,000 IU per day yields no additional calcemic benefit [125]. This diminished responsiveness indicates that endogenous negative feedback networks or a rate-limiting constraint in renal CYP27B1 activation restricts the therapeutic efficacy of unactivated vitamin D_3_ form [113]. In small ruminants, the timing of administration is equally critical, as a single intramuscular dose of 10,000 IU of vitamin D in mid-pregnancy has been shown to prevent gestational decline and significantly elevate postpartum serum calcium and phosphorus levels. Furthermore, this mid-gestation intervention optimizes maternal energy balance by lowering beta-hydroxybutyrate and non-esterified fatty acid concentrations while enhancing postpartum protein synthesis [127].

In addition, intravenous co-administration of 1α-hydroxyvitamin D_3_ and 25-hydroxyvitamin D_3_ has demonstrated a robust capacity to elevate plasma 1,25(OH)_2_D_3_ levels throughout the calving period, successfully raising serum calcium by approximately 2 mg/dL [146]. Similarly, direct parenteral administration of 300 μg of calcitriol at six hours postpartum provides a safe and effective calcemic response, maintaining elevated serum calcium concentrations during the first six days postpartum and reducing the prevalence of subclinical hypocalcemia from 19.0% to 9.3% in treated cohorts [147].

Despite these prophylactic merits in mitigating subclinical deficits, active vitamin D analogs exhibit limited utility as an acute curative intervention for clinical PP. Injectable vitamin D formulations cannot serve as a standalone replacement for rapid intravenous calcium infusions in recumbent animals. Instead, their primary clinical utility resides in strategic prepartum or immediate postpartum prophylaxis to minimize homeostatic insufficiency during the transition phase.

### 6.5. Prepartum 5-Hydroxytryptophan Supplementation Modulating the 5-HT-PTHrP–Bone Axis

Prepartum administration of 5-HTP serves as a targeted strategy to preemptively bolster postpartum calcium homeostasis by stimulating the serotonergic pathway. In multiparous Holstein cows, daily intravenous infusions of 1.0 mg/kg of 5-HTP initiated approximately seven days prior to the expected calving date face a significant upregulation in the mRNA expression of the *CaSR* during early lactation on days 1 and 7 [128,129]. This molecular upregulation enhances the sensitivity of the sensing mechanisms that govern peripheral calcium mobilization.

This regulatory pattern is supported by physiological profiles in small ruminants, such as Guanzhong dairy goats, which naturally exhibit a concurrent progressive decline in both serum serotonin and calcium concentrations from day 3 relative to parturition until the day of kidding [85]. Prophylactic prepartum elevation of systemic serotonin concentrations effectively counteracts this periparturient depletion. By reinforcing the 5-HT-PTHrP–bone axis prior to the acute metabolic stress of lactogenesis, 5-HTP administration stabilizes postpartum calcemia and reduces PP susceptibility.

### 6.6. Genomic Selection and Multi-Omic Integration for Hypocalcemia Resistance

Genomic selection leverages markers across the entire genome to capture quantitative trait loci in linkage disequilibrium, providing a high-resolution framework for breeding disease-resistant livestock [148]. However, the genetic improvement of transition health has historically been bottlenecked by the low heritability estimates of clinical disorders. Recent large-scale population studies and comprehensive meta-analyses confirm that PP exhibits a true threshold heritability centered tightly around 0.03 to 0.07 [21,23]. This low coefficient is largely driven by the limitations of traditional binary health logging, which collapses continuous biological variation into a simplistic categorical state and fails to capture the true underlying genetic variance. This statistical challenge is heavily compounded by the widespread underreporting of SCH. By failing to record cows experiencing severe transient drops in blood calcium without recumbency, reference populations suffer from severe phenotypic misclassification. Recent initiatives demonstrate that shifting toward continuous indicators—such as utilizing real-time serological calcium curves or ionized calcium fractions as phenotypic targets—significantly refines genomic breeding value estimation [149,150].

Modern genome-wide association studies (GWAS) and gene-set analyses reveal a highly polygenic architecture for calcemic stability, with critical variants predominantly clustered within regions governing calcium channel function, bone remodeling, and intracellular signaling pathways [21,22,130]. While advanced mixed-model association tests have validated specific candidate genes directly linked to cow livability and hypocalcemia resistance [151], recent structural genomics has identified CNV regions associated with metabolic health that alter the gene dosage of key homeostatic networks [152]. To capture this polygenic and structural variance, standard GBLUP has been widely superseded by ssGBLUP, which unifies pedigree, genotypes, and ungenotyped herd records into a single matrix [23]. Advanced Bayesian alphabet models are concurrently deployed to accommodate unequal genetic variances across chromosomal regions, yielding stable genomic prediction accuracies between 0.25 and 0.42 [23].

Furthermore, breeding progress is influenced by significant genetic correlations with production traits. Intensive genetic selection focused solely on maximizing milk volume shares an unfavorable genetic correlation with periparturient calcium stability, meaning that cows with superior genetic merit for production are inherently predisposed to homeostatic failure at the onset of lactation [153]. To resolve these antagonistic genetic correlations, modern dairy breeding strategies employ comprehensive multi-trait selection indices rather than selecting for single health traits. These selection indices balance metabolic resilience with profitable production by weighting wellness traits and hypocalcemia resistance directly alongside economic components and yield traits, allowing producers to apply balanced selection pressure that maintains health without sacrificing performance [131,133].

Nevertheless, three critical field limitations constrain the efficiency of this genomic framework. First, phenotypic pipeline gaps—specifically the absence of standardized, automated diagnostic tracking for subclinical metabolic disorders—severely limit the size and reliability of genomic reference populations [23]. Second, cross-breed accuracy decay rapidly degrades the predictive performance of genomic models when applied across genetically distant breeds or crossbred systems due to inconsistent linkage disequilibrium blocks. Finally, genomic inbreeding depression presents an ongoing risk, as intensive selection for elite genomic lines accelerates autozygosity; indeed, genomic analyses utilizing Runs of Homozygosity reveal that rapid inbreeding is positively correlated with elevated incidences of metabolic disorders due to inbreeding depression in fitness traits [154].

To circumvent these operational barriers, the management of PP increasingly integrates genomic markers within multi-omic frameworks alongside phenotypic production data and longitudinal metabolomic profiles [132]. Overlaying an animal’s inherited genomic liability with real-time metabolic monitoring enables sensitive, machine-learning-driven predictive models to identify high-risk individuals prior to clinical onset. This integration allows producers to deploy targeted, individual-level preventive measures, bridging quantitative genetics and precision livestock management to preserve long-term herd metabolic stability.

## 7. Conclusions and Future Directions

Parturient paresis represents a systemic, multi-organ endocrine and homeorhetic failure to compensate for the acute calcium clearance demands triggered by early lactogenesis. The underlying pathogenesis involves complex dysregulation across the parathyroid hormone–vitamin D, 5-HT–PTHrP, and FGF23 axes, characterized by peripheral receptor resistance and delayed enzymatic response kinetics. Although herd management strategies have successfully progressed from reactive parenteral interventions to proactive nutritional priming and systemic acid–base modulation, long-term mitigation requires moving beyond group-level dietary adjustments toward resolving fundamental molecular discrepancies within these intersecting networks.

To advance transition cow biology, future research must prioritize validating the functional mechanics of emerging regulatory pathways directly within periparturient ruminants. Critical knowledge gaps remain regarding the precise temporal expression and systemic triggers of FGF23 during acute calcemic shifts, which are vital to understanding delayed renal activation. Similarly, the exact timing of mammary-derived serotonin receptor transactivation requires structural validation to optimize bone resorption without inducing systemic feedback inhibition. Furthermore, clarifying the role of the microbiota-gut–calcium axis—specifically how microbial metabolites modulate epithelial transport channels like TRPV6 and tight-junction claudins—will uncover new opportunities for targeted gastrointestinal interventions to enhance both passive and active mineral absorption.

Ultimately, bridging molecular endocrinology with quantitative genetics offers a sustainable framework to breed resilient livestock and achieve precision prevention. Transitioning to advanced single-step genomic BLUP architectures and comprehensive multi-trait economic selection indices allows breeding programs to build permanent genetic resistance, effectively mitigating the antagonistic genetic correlation between high milk yield and homeostatic stability. Integrating these heritable genomic liabilities with real-time longitudinal metabolomic profiles and machine-learning models enables producers to deploy targeted, individual-level preventive measures. This holistic framework will transition herd management from reactive treatments to predictive prophylaxis, ultimately reducing disease incidence while enhancing the production efficiency, health, and welfare of high-yielding dairy herds.

## Figures and Tables

**Table 2:** Comparative Matrix of Hypocalcemia Prevention and Treatment Interventions.

Management	Mechanisms of Action	Stage of Application	Major Advantages	Limitations	Level of Evidence	Reference
Intravenous Calcium	Rapidly restores extracellular ionized calcium pool via direct vascular infusion	Acute clinical phase at the immediate onset of recumbency	Immediate life-saving therapeutic rescue for paretic animals	Severe risk of fatal cardiotoxicity if infused too rapidly; triggers calcitonin release causing rebound hypocalcemia	High; universally accepted clinical gold standard	[107,108,109]
Oral Calcium Supplements	Promotes paracellular passive intestinal absorption driven by high luminal concentration gradients	Postpartum period within the first 24 h after calving	Extremely safe; avoids cardiac risks; provides a sustained release profile over 6 to 12 h	Completely ineffective for recumbent or paretic cows; risk of aspiration pneumonia if drenched improperly	High; extensively validated across commercial dairy systems	[110,111,112]
Dietary Cation–Anion Difference	Induces mild compensated metabolic acidosis to optimize tissue PTH1R stereochemical configuration	Close-up dry period spanning 14 to 21 days prepartum	Exceptional herd-level control; reduces clinical incidence by up to 87 percent	Completely ineffective for recumbent or paretic cows; risk of aspiration pneumonia if drenched improperly	High; standard industry benchmark for prepartum prevention	[75,78,113,114,115,116]
Synthetic Zeolite	Non-selectively binds dietary calcium within the gastrointestinal tract to force prepartum PTH activation	Close-up dry period spanning 14 to 21 days prepartum	Highly effective prevention without altering systemic acid–base balance	Requires exceptionally high dietary inclusion rates; expensive; risks binding other essential trace minerals	Moderate to High; well-supported by recent transition trials	[117,118,119,120,121]
Vitamin D Derivatives	Bypasses renal hydroxylation to directly transactivate enterocyte TRPV6 and Calbindin expression	Prepartum window targeting 2 to 8 days prior to expected calving date	Extremely potent biological upregulation of active transcellular transport pathways	Extremely narrow safety margin; massive risk of hypervitaminosis and tissue calcification if calving is delayed	Moderate; inconsistent clinical efficacy due to unpredictable calving dates	[122,123,124,125,126,127]
5-Hydroxytryptophan	Stimulates localized mammary TPH1 expression to upregulate systemic bone-mobilizing PTHrP	Late pregnancy extending into the early postpartum transition phase	Promising new physiological vector targeting the natural mammary calcium sensor network	Currently restricted to experimental research models; optimal commercial dosages and long-term impacts remain unvalidated	Moderate; emerging biological framework requiring field-scale validation	[128,129]
Genomic Selection	Selects for heritable traits linked to elevated baseline target-tissue VDR density and structural resilience	Long-term herd breeding and genetic selection strategies	Permanent, cumulative genetic progress; non-invasive and eliminates daily management friction	Exceptionally slow generational interval; complex, multi-trait correlations with absolute milk yield can complicate selection indexes	Moderate; emerging epidemiological integration with long-term tracking	[21,23,130,131,132,133]

## Data Availability

No new data were created or analyzed in this study. Data sharing is not applicable to this article.
